# The Cost-Utility Analysis of PET-Scan in Diagnosis and Treatment of Non-Small Cell Lung Carcinoma in Iran

**DOI:** 10.5812/iranjradiol.8559

**Published:** 2013-05-20

**Authors:** Ali Akbari Sari, Hamid Ravaghi, Mohammadreza Mobinizadeh, Sima Sarvari

**Affiliations:** 1 Deptartment of Health Management and Economics, Knowledge Utilization Research Center, Tehran University of Medical Sciences, Tehran, Iran; 2 School of Health Management and Information Sciences, Department of Health Service Management, Tehran University of Medical Sciences, Tehran, Iran; 3 Department of Health Service Management, School of Management and Economics, Science and Research Branch, Islamic Azad University, Tehran, Iran; 4 Department of Medicine, Shantou University Medical College, Shantou, China

**Keywords:** Positron-Emission Tomography, Non-Small-Cell Lung Carcinoma, Economics

## Abstract

**Background:**

PET scan is a non-invasive, complex and expensive medical imaging technology that is normally used for the diagnosis and treatment of various diseases including lung cancer.

**Objectives:**

The purpose of this study is to assess the cost effectiveness of this technology in the diagnosis and treatment of non- small cell lung carcinoma (NSCLC) in Iran.

**Materials and Methods:**

The main electronic databases including The Cochrane Library and Medline were searched to identify available evidence about the performance and effectiveness of technology. A standard decision tree model with seven strategies was used to perform the economic evaluation. Retrieved studies and expert opinion were used to estimate the cost of each treatment strategy in Iran. The costs were divided into three categories including capital costs (depreciation costs of buildings and equipment), staff costs and other expenses (including cost of consumables, running and maintenance costs). The costs were estimated in both IR-Rials and US-Dollars with an exchange rate of 10.000 IR Rials per one US Dollar according to the exchange rate in 2008.

**Results:**

The total annual running cost of a PET scan was about 8850 to 13000 million Rials, (0.9 to 1.3 million US$). The average cost of performing a PET scan varied between 3 and 4.5 million Rials (300 to 450US$). The strategies 3 (mediastinoscopy alone) and 7 (mediastinoscopy after PET scan) were more cost-effective than other strategies, especially when the result of the CT-scan performed before PET scan was negative.

**Conclusion:**

The technical performance of PET scan is significantly higher than similar technologies for staging and treatment of NSCLC. In addition, it might slightly improve the treatment process and lead to a small level of increase in the quality adjusted life year (QALY) gained by these patients making it cost-effective for the treatment of NSCLC.

## 1. Background

There has been a rapid increase in the use of medical technologies in the recent years. It is clear that proper use of these technologies can significantly improve the patients’ conditions; however, uncontrolled and inappropriate use of them might lead to a waste of limited resources (1). PET scan is a non-invasive, expensive medical imaging technology that was introduced in 1950. This technology is currently used for diagnosis and treatment of various diseases, especially cancers across the world (2-4). Compared to the MRI and CT scan that explore the cancer lesions at an anatomical level, this technology can explore the cancer lesions at cellular and biochemical levels that might lead to a higher diagnostic performance (3). Various countries have commonly used PET scan for staging and treatment of cancers, especially lung cancers and non small cell lung carcinoma (NSCLC). Approximately 85% to 90% of lung cancers are non-small cell lung carcinoma (NSCLC) (2). Recent studies from the UK and Australia have shown that the technology is significantly cost-effective, particularly when it is used for staging and treatment of NSCLC and lymphoma (3, 5). It is argued that the evidence regarding the technical performance and effectiveness of technologies are generalizable between the countries, but the cost-effectiveness evidence cannot be generalized to other countries (6).

## 2. Objectives

The main purpose of this research is to explore the cost effectiveness of PET scan in staging and treatment of NSCLC in Iran that might then help optimize the allocation of limited resources.

## 3. Materials and Methods

The process of using PET scan in staging lung cancer: PET scan is not normally used for screening and diagnosis of lung cancer. When a patient is diagnosed with lung cancer and the biopsy shows that the cancer is NSCLC, then normally a CT scan is performed to explore any metastasis (3). The accuracy of CT scan for detecting the cancer metastasis is moderate and performing a PET scan at this stage can improve the accuracy of staging (3). The accuracy of PET scan at this stage is different depending on the result of the CT scan, whether it is positive or negative. In addition, according to the marker studies (3, 5), there are seven possible strategies for detecting metastasis in NSCLC after CT scan. Therefore, for the purpose of economic evaluation, we designed a standard decision tree model that included seven strategies after the CT scan was negative and seven strategies after the CT scan was positive consisting of a total of 14 strategies (Table 1).

**Table 1.** Seven Major Strategies Used in the Modeling of Cost Effectiveness

Strategy	Explanation
1	Sending all patients for surgery
2	Sending all patients for non-surgical treatment
3	All patients were examined by mediastinoscopy
	a. If mediastinoscopy is negative, surgery will be done
	b. If mediastinoscopy is positive, non-surgical treatment will be done
4	All patients were examined by mediastinoscopy
	a. If mediastinoscopy is negative, PET scan will be done
	1) If FDG-PET is negative, surgery will be done
	2) If FDG-PET is positive, non-surgical treatment will be done
	b. If mediastinoscopy is positive, nonsurgical treatment will be done
5	All patients were examined by FDG-PET
	a. If FDG-PET is negative, surgery will be done
	b. If FDG-PET is positive, non-surgical treatment will be done
6	All patients were examined by FDG-PET
	a. If FDG-PET is negative, mediastinoscopy will be done
	1)If mediastinoscopy is negative, surgery will be done
	2) If mediastinoscopy is positive, non-surgical treatment will be done
	b. If FDG-PET is positive, non-surgical treatment will be done
7	All patients were examined by FDG-PET
	a. If FDG-PET is negative, surgery will be done
	b. In the presence of distant metastasis, nonsurgical treatment will be done
	c. In the presence of metastasis in the mediastinal area, mediastinoscopy will be done
	1) If mediastinoscopy is negative, surgery will be done
	2) If mediastinoscopy is positive, non-surgical treatment will be done

The data about technical and clinical performance of PET scan were not available in Iran, because the technology had not yet arrived to the country. Therefore, the main electronic databases including Cochrane Library (Issue 4, 2008) and Medline (Nov 2008) were searched to identify any systematic reviews, economic evaluations and/or health technology assessments published in this area. The quality of retrieved reports was checked using standard CRD (Center for Review and Dissemination) criteria (7). Those reports that met the quality criteria and were up to date and also had the potential for answering parts or all of the study questions were included. Iranian national databases including IranMedex and SID were searched to identify local and national socio-demographic data and data about the incidence of lung cancer and NSCLC, the staging of disease at the time of diagnosis and other local data required for the model. Retrieved studies and expert opinion were used to estimate the cost of each treatment strategy in Iran. The costs were divided into three categories including capital costs (depreciation costs of buildings and equipment), staff costs and other expenses (including cost of consumables, running and maintenance costs). The costs were estimated in both IR-Rials and US-Dollars with an exchange rate of 10.000 IR Rials per one US Dollar according to the exchange rate in 2008. Sensitivity analysis was used to explore any possible uncertainties in the socio-demographic and costing data. The data about the costs of each strategy were collected according to the local tariffs and expenses in Iran at the time of study. Sensitivity analysis was performed according to the following assumptions. These assumptions were made according to the current situation at the time of the study either from the robust evidence or expert opinions. Assumption A: 3% of patients had distant metastasis, 7% had distant and mediastinal metastasis, 27% had mediastinal metastasis and 63% of patients had local lesions with no metastasis (3). Assumption B: 4% of patients had distant metastasis, 7% had distant and mediastinal metastasis, 24% had mediastinal metastasis and 65% of patients had local lesions with no metastasis (8). Assumption C: 20% of patients had distant metastasis, 10% had distant and mediastinal metastasis, 50% had mediastinal metastasis and 20% of patients had local lesions with no metastasis. Assumption D: 20% of patients had distant metastasis, 20% had distant and mediastinal metastasis, 20% had mediastinal metastasis and 10% of patients had local lesions with no metastasis (Table 2).

**Table 2.** Different Assumptions about the Early Detection of NSCLC in Iran

Grading Cancer	Spread of Cancer	Option A (%)	Option B (%)	Option C (%)	Option D (%)
N0/1M0	Local	63	65	20	10
N0/1M1	Distant Metastasis	3	4	20	20
N2/3M0	Regional Metastasis	27	24	50	20
N2/3M1	Both	7	7	10	20
Total		100	100	100	100

Abbreviations: M, metastasis; N, node

## 4. Results

The total costs of setting up a complete PET scan unit were 81600 million Rials (8.2 million US$). The total costs were 57000 million Rials (5.7 million US$) when we excluded the building costs and 37400 million Rials (3.7 million US$) when we excluded the building costs and the costs of the cyclotron unit (Tables 3, 4, 5 and 6). Assuming that a PET scan will perform about 3000 tests a year, the total annual cost of running a PET scan unit including building, equipment, staff and consumable products were 10860 to 13000 million Rials (1.08 to 1.3 million US$). Excluding the building costs, the total annual cost of running a PET scan were 9430 million to 11000 million Rials (0.9 to 1.1 million US$). Excluding the building costs and purchasing the FDG-18 from another cyclotron unit, the total annual cost of running a PET scan was 8850 to 10900 million Rials (0.8 to 1.09 million US$). Considering the above mentioned assumptions, the cost of a PET scan test was 3,000,000 to 4,400,000 Rials (300 to 440 US$). Assuming a discount rate of zero and a 10% increase in staff salary, the total projected costs of running a complete PET scan unit were about 28 trillion Rials (2800 million US$) after 20 years (Tables 3, 4, 5 and 6). Table 7 shows the minimum and maximum local costs of each single intervention that we used to estimate the costs of each strategy.

**Table 3.** Projected Costs of PET Facility in Iran (Option 1)

Full Unit in Iran, IRR	Total Capital Costs, IRR	Annual Costs, IRR	Annual lower Costs, IRR	Life, y	Scan/yr	Cost/Scan (High), IRR	Cost/Scan (Low), IRR
Scanner	35,000,000,000	4,486,965,000	4,486,000,000	10	3,000	1,495,655	1,495,333
Comp Eq	400,000,000	89,502,400	60,000,000	5	3,000	29,834	20,000
Scan Eq	2,000,000,000	256,398,000	123,000,000	10	3,000	85,466	41,000
Building	9,000,000,000	737,100,000	600,000,000	30	3,000	245,700	200,000
Cyclotron	15,000,000,000	1,234,646,250	1,200,000,000	20	3,000	411,549	400,000
Cycl Lab Eq	5,000,000,000	640,995,000	400,000,000	10	3,000	213,665	133,333
Cycl Comp Eq	200,000,000	44,880,000	30,000,000	5	3,000	14,960	10,000
Cycl Build	15,000,000,000	1,228,500,000	840,000,000	30	3,000	409,500	280,000
Total Capital	81,600,000,000	8,718,986,650	7,739,000,000		3,000	2,906,329	2,579,667
Staff PET		1,410,000,000	965,000,000		3,000	470,000	321,667
Staff Cycl		430,000,000	285,000,000		3,000	143,333	95,000
Staff Total		1,840,000,000	1,250,000,000		3,000	613,333	416,667
PET Maint		200,000,000	150,000,000		3,000	66,667	50,000
Comp Maint		20,000,000	15,000,000		3,000	6,667	5,000
Housekeeping		30,000,000	20,000,000		3,000	10,000	6,667
Marketing/Training		30,000,000	20,000,000		3,000	10,000	6,667
Rod		30,000,000	20,000,000		3,000	10,000	6,667
Power/Building Maint		20,000,000	15,000,000		3,000	6,667	5,000
Total PET Maint		330,000,000	240,000,000		3,000	110,000	80,000
Cycl Maint		150,000,000	100,000,000		3,000	50,000	33,333
Lab Maint		40,000,000	30,000,000		3,000	13,333	10,000
Housekeeping		40,000,000	40,000,000		3,000	13,333	13,333
Power/Building Maint		30,000,000	30,000,000		3,000	10,000	10,000
Total Cycl Maint		260,000,000	200,000,000		3,000	86,667	66,667
Scanning Supplies (3000)		300,000,000	250,000,000		3,000	100,000	83,333
Data Copy		40,000,000	40,000,000		3,000	13,333	13,333
Hard Copy and Storage		120,000,000	100,000,000		3,000	40,000	33,333
Total PET Variables		460,000,000	390,000,000		3,000	153,333	130,000
Lab Supplies		500,000,000	350,000,000		3,000	166,667	116,667
Chemicals/Target Materials		600,000,000	500,000,000		3,000	200,000	166,667
Gases		300,000,000	200,000,000		3,000	100,000	66,667
Total Cycl Variables		1,400,000,000	1,050,000,000		3,000	466,667	350,000
Total Costs		13,008,986,650	10,869,000,000		3,000	4,336,329	3,623,000

Abbreviations: Eq, equipment; Cycl, Cyclotron; Maint, maintenance; IRR, Iranian Rial; y, year

**Table 4.** Projected Costs of PET Facility in Iran (Option 2)

Full Unit with Free Building Capital Costs	Total Capital Costs, IRR	Annual Costs, IRR	Annual Lower Costs, IRR	Scan/yr	Cost/Scan (High), IRR	Cost/Scan (Low), IRR
Total Capital	57,600,000,000	6,753,386,650	6,299,000,000	3,000	2,251,129	2,099,667
Total Staff		1,840,000,000	1,250,000,000	3,000	613,333	416,667
Total PET Maint		330,000,000	240,000,000	3,000	110,000	80,000
Total Cycl Maint		260,000,000	200,000,000	3,000	86,667	66,667
Total Cycl Variables		1,400,000,000	1,050,000,000	3,000	466,667	350,000
Total Costs		11,043,386,650	9,429,000,000	3,000	3,681,129	3,143,000

Abbreviations: Cycl, Cyclotron; Maint, maintenance; IRR, Iranian Rial

**Table 5.** Projected Costs of PET Facility in Iran (Option 3)

PET Unit, No Building Capital Cost, No Cyclotron Unit	Total Capital Costs, IRR	Annual Costs, IRR	Annual Lower Costs, IRR	Scan/yr	Cost/Scan (High), IRR	Cost/Scan (Low), IRR
Total Capital	37,400,000,000	4,832,865,400	4,669,000,000	3,000	1,610,955	1,556,333
Total Staff		1,410,000,000	965,000,000	3,000	470,000	321,667
Total PET Maint		330,000,000	240,000,000	3,000	110,000	80,000
Total Cycl Maint		0	0	3,000	0	0
Total PET Variables		460,000,000	390,000,000	3,000	153,333	130,000
Total Cycl Variables		0	0	3,000	0	0
Total Cycl Purchase		3,869,482,222	2,590,000,000	3,000	1,289,827	863,333
Total Costs		10,902,347,622	8,854,000,000	3,000	3,634,116	2,951,333

Abbreviations: Cycl, Cyclotron; Maint, maintenance IRR, Iranian Rial

**Table 6.** Projected Costs of a Cyclotron Facility in Iran

Cyclotron for 6000 Scans, Plus 3000 Delivery	Total Capital Costs, IRR	Annual Costs, IRR	Annual lower Costs, IRR	Scan/yr	Cost/Scan (High), IRR	Cost/Scan (Low), IRR
Total Capital	35,200,000,000	3,668,964,444	2,470,000,000	6,000	611,494	411,667
Staff Cycl Total		600,000,000	400,000,000	6,000	100,000	66,667
Total Cycl Maint		370,000,000	260,000,000	6,000	61,667	43,333
Total Cycl Variables		2,800,000,000	1,900,000,000	6,000	466,667	316,667
Total Cost of Cycl		7,738,964,444	5,180,000,000	6,000	1,289,827	863,333

Abbreviations: Cycl, Cyclotron; Maint, maintenance; IRR, Iranian Rial

**Table 7.** Different Assumptions About the Costs in Iran

Costs in the Model, IRR	Low Costs	Costs, mean	High Costs	Very High Costs
Options for Which the Costs Were Used	Option A	Option B	Option C	Option D
Surgery, mean	8,000,000	10,000,000	12,000,000	20,000,000.00
Chemotherapy	7,000,000	7,500,000	8,000,000	10,000,000.00
Radiotherapy	7,000,000	7,500,000	8,000,000	10,000,000.00
Mediastinoscopy	1,500,000	1,750,000	2,000,000	3,500,000.00
PET	4,000,000	5,000,000	6,000,000	7,000,000.00

Abbreviations: IRR, Iranian Rial

Option A - (low cost and low incidence of metastasis): Table 8 shows that when the CT scan was positive, the third strategy had the lowest cost per QALY followed by the first, fourth, seventh, fifth, sixth and second strategies. In addition, when the CT scan was negative, the first strategy had the lowest cost per QALY, followed by the third, seventh, fifth; fourth, sixth and second strategies. Option D - (high costs and the high incidence of metastasis): when the CT scan results were positive: the third strategy would be associated with the lowest cost per QALY and the fourth; sixth, fifth, second, seventh and the first strategy would be placed in the next steps in an increasing order. When the CT scan results were negative: the third strategy would be associated with the lowest cost per QALY and the fourth; sixth, fifth, seventh, first and second strategy would be placed in the next steps in an increasing order. The incremental cost effectiveness ratio (ICER) for each strategy was calculated for options A and D, presented in Tables 9 and 10.

**Table 8.** The Cost Effectiveness of Various Strategies Using PET Scan Technology

	Strategies	The Cost Per QALY for Option A, IRR	The Cost per QALY for Option B, IRR	The cost per QALY for the Option C, IRR	The cost per QALY for Option D, IRR
1	1. 1 , CT+	9,355,666	11,510,392	11,026,581	17,344,249
	1. 2 , CT-	4,873,201	7,007,086	7,783,681	12,804,755
2	2. 1 , CT+	14,756,475	14,955,508	14,197,408	14,214,559
	2. 2 , CT-	13,990,576	14,083,853	14,836,450	14,854,692
3	3. 1 , CT+	8,456,866	9,995,084	9,424,489	12,436,273
	3. 2 , CT-	4,992,172	6,472,773	6,869,124	9,976,253
4	4. 1 , CT+	10,072,166	10,544,595	9,880,870	12,507,749
	4. 2 , CT-	6,865,475	7,523,883	7,805,994	10,880,882
5	5. 1 , CT+	11,063,261	12,365,090	11,683,178	13,895,080
	5. 2 , CT-	6,617,244	8,002,483	8,349,699	11,124,347
6	6. 1 , CT+	11,519,007	12,456,886	11,736,912	13,868,767
	6. 2 , CT-	7,028,222	8,152,054	8,427,113	11,093,117
7	7. 1 , CT+	10,449,837	12,221,175	11,459,729	15,260,508
	7. 2 , CT-	6,227,582	7,929,976	8,228,064	11,837,700

Abbreviation: QALY, quality adjusted life year; IRR, Iranian Rial

**Table 9.** The ICER for Option A for Each of the Seven Strategies

Strategies	Additional Costs Compared to the Cheapest Strategy, IRR	Additional QALY Compared to the Reference (Cheapest) Strategy	ICER
Strategy 3, CT+	-(Cheapest strategy; total cost=606,176,673)	- (Total QALY=71.68)	-
Strategy 1, CT+	45,173,038	-2.06	Dominated by 3
Strategy 2, CT+	51,737,840	-27.10	Dominated by 3
Strategy 4, CT+	57,058,552	-137.53	Dominated by 3
Strategy 5, CT+	126,114,721	-5.49	Dominated by 3
Strategy 7, CT+	146,437,292	0.34	430,697,918
Strategy 6, CT+	9,843,856	-5.49	Dominated by 7
Strategy 1, CT-	-(Cheapest strategy; total cost=922,143,745)	- (Total QALY=189.23)	-
Strategy 3, CT-	25,792,563	0.65	39,680,866
Strategy 7, CT-	268,561,423	1.97	136,325,595
Strategy 5, CT-	13,633,874	-7.23	Dominated by 7
Strategy 4, CT-	54,300,961	-7.89	Dominated by 7
Strategy 6, CT-	83,813,918	-7.89	Dominated by 7
Strategy 2, CT-	151,380,548	-93.30	Dominated by 7 and 6

Abbreviation: QALY, quality adjusted life year; ICER, incremental cost effectiveness ratio; IRR, Iranian Rial

**Table 10.** The ICER for Each of the Seven Strategies for Option D

Strategies	Additional Costs Compared to the Cheapest strategy, IRR	Additional QALY Compared to the Reference Strategy, IRR	ICER
Strategy 2, CT+	-(Cheapest strategy; total cost=971,991,584)	-(Total QALY=68.38)	-
Strategy 4, CT+	291,191,052	32.61	8,929,502
Strategy 3, CT+	395,177,620	41.55	9,510,893
Strategy 6, CT+	435,937,922	33.14	13,154,433
Strategy 5, CT+	438,609,205	33.14	13,235,039
Strategy 7, CT+	713,684,164	42.08	16,960,175
Strategy 1, CT+	879,992,636	38.40	22,916,475
Strategy 2, CT-	-(Cheapest strategy; total cost=1,028,004,124)	- (Total QALY=66.2)	-
Strategy 3, CT-	338,602,822	67.79	4,994,879
Strategy 4, CT-	395,476,409	61.62	6,417,988
Strategy 6, CT-	423,241,750	61.62	6,868,578
Strategy 5, CT-	432,600,379	62.10	6,966,190
Strategy 7, CT-	604,817,207	68.73	8,799,901
Strategy 1, CT-	719,998,578	67.31	10,696,755

Abbreviation: QALY, quality adjusted life year; ICER, incremental cost effectiveness ratio; IRR, Iranian Rial

## 5. Discussion

We found that strategies 3 (mediastinoscopy alone) and 7 (mediastinoscopy after PET scan) were cost-effective in Iran, especially when the CT scan result was negative. Assuming that a large proportion of Iranian patients might be diagnosed with metastasis in whom surgery cannot be performed, the fourth strategy (PET scan after mediastinoscopy) be more cost effective than the other strategies, especially when the CT scan was negative. Two economic models published in the UK in 2002 and 2007 (3, 9) showed that when diagnosis of NSCLC was confirmed with conventional methods such as biopsy and CT scan, the use of PET-FDG in assessing the degree of malignancy before surgery was cost effective, especially in those patients who had a negative CT scan. The Scottish study indicated that the use of PET scan is effective only if the willingness to pay is higher than 60,000 pounds per QALY (9). Another study was published in 2003 in Australia showing that use of PET scan leads to an average increase of 0.046 years of patient life (about 17 days) and an average increase of $189 in cost per each patient. This means that the additional cost per additional QALY gained is about $ 41.087 (5). A third study was conducted in 2002 in Scotland that used a decision model with 5 strategies for the evaluation of PET. This study showed that using CT scan without PET scan leads to an increase in life expectancy and reduction in cost. But this approach leads to radiotherapy and unnecessary treatment in 36 percent of the patients. In contrast, when the technology alone and without FDG-PET scans was used, this figure reduced to 4%. In addition, it led to 0.7 increase in the average years and 236 pounds reduction in the cost per patient (10). Considering the small changes of this technology in the clinical and final outcomes (QALY) of the patients and its small savings costs, the final decision about using or not using this technology in Iran depends on whether the technical performance of the test is considered more important or the clinical final outcomes. If early and more accurate detection of the disease is more important than the final outcomes, then the technology should be considered useful. PET scan seems to be an effective technology and could be diffused in Iran based on a comprehensive technology rationing system at national and regional levels. To increase the efficiency and preventing induced demand, the indication for use of this technology in Iran should be NSCLC. Based on available information, some Iranian medical centers received the license for entering PET scan, but only two of them could enter this technology with attention to this point that PET scan has not been installed completely in these centers yet. The actual costs and clinical findings of PET scan were not available in Iran due to the unavailability of the technology, so the data were estimated based on international evidence. Drummond et al. argued that the clinical data can be used and generalized between the countries (6). To minimize the possible bias, we used the socio-demographic and costing data according to the local evidence available in Iran.
